# Hidden risk: Latent cognitive profiles and structural brain age reveal vulnerability in midlife metabolic syndrome

**DOI:** 10.1017/S1355617725101604

**Published:** 2025-11

**Authors:** Isabelle Gallagher, Makenna B. McGill, Janelle T. Foret, Hirofumi Tanaka, David M. Schnyer, Andreana P. Haley

**Affiliations:** 1 Department of Psychology, The University of Texas at Austin, Austin, TX, USA; 2 Department of Kinesiology and Health Education, The University of Texas at Austin, Austin, TX, USA

**Keywords:** adult, cardiovascular diseases, humans, triglycerides, aging, cognitive dysfunction

## Abstract

**Objective:**

Metabolic syndrome (MetS) is linked to later-life cognitive decline and brain aging, but early detection of vulnerability in midlife remains challenging. This study applied two methods to detect subtle changes in midlife adults with MetS: (1) latent profile analysis (LPA) to identify cognitive performance patterns and (2) an MRI-derived brain-predicted age metric to assess structural brain aging.

**Method:**

Participants were cognitively unimpaired, community-dwelling adults from prior studies on metabolic and brain health (*N* = 230; ages 40 – 65). MetS status was assigned using clinical criteria based on cardiovascular indicators and medical history. Cognitive test scores, adjusted for age, sex, and education, were analyzed using LPA, identifying four cognitive subgroups: High Memory, Low Executive, Global Average, and Low Memory. T1-weighted MRI scans were processed with brainageR to compute brain-predicted age difference (PAD). Analyses were conducted in R using chi-square tests, ANCOVA, regression, and nonparametric methods, with appropriate covariates and effect size estimates.

**Results:**

MetS prevalence differed across cognitive profiles (χ^2^ = 10.99, *p* = .012, *V* = 0.22), with higher rates in the Low Memory and Global Average groups than in the High Memory group. Individuals without MetS had younger brain ages than those with MetS (*p* = 0.003, η^2^ = 0.03). Only elevated triglycerides were associated with greater PAD (*p* = 0.012, η^2^ = 0.02). A Johnson–Neyman analysis showed the MetS–PAD association was significant between ages 40.0 and 54.6. PAD did not differ by cognitive profile.

**Conclusions:**

Cognitive profiles and brain-predicted age metrics identify early vulnerability in midlife MetS, underscoring the importance of early intervention.

## Statement of Research Significance

**Research Question(s) or Topic(s):** This study examined patterns of cognitive functioning and structural brain aging in 230 cognitively healthy, middle-aged adults with and without metabolic syndrome. **Main Findings:** Adults with metabolic syndrome were more likely to show cognitive patterns of poorer memory and average cognitive performance. In contrast, those without metabolic syndrome had brain-predicted ages approximately 2.5 years younger than their counterparts and were more likely to show cognitive patterns of better memory performance. The link between metabolic syndrome and older brain age was strongest in early midlife. **Study Contributions:** This study shows that subtle differences in memory and brain aging can be detected in middle age, even among adults without cognitive symptoms. It highlights the value of methods that group people by cognitive patterns and estimate brain aging from structural brain scans. Findings suggest that early midlife is a key window for preventing cardiovascular conditions that may raise the risk for memory decline and dementia later in life.

## Introduction

Metabolic Syndrome (MetS) is a cluster of well-established risk factors for later-life cognitive decline and dementia (Qureshi et al., 2024). MetS is defined by the presence of three or more metabolic health conditions, including abdominal obesity, hypertension, elevated triglycerides, reduced HDL cholesterol, and elevated fasting glucose concentrations (Alberti et al., 2009). Midlife cardiovascular and metabolic health conditions are strongly associated with increased risk for dementia in later life (Beeri & Gu, 2024; Livingston et al., 2020; Whitmer et al., 2005). Recent findings emphasize that both the timing and chronicity of metabolic burden may be particularly important. Midlife multimorbidity, having two or more chronic health conditions (including cardiometabolic health conditions), is associated with more than double the risk of future dementia, with risk increasing further in cases of earlier onset or greater severity of disease (Ben Hassen et al., 2022).

There is growing recognition of the link between metabolic dysregulation and subtle cognitive dysfunction across domains such as memory, executive functioning, and processing speed in both middle- and older-aged adults (Falkowski et al., 2014; Foret et al., 2021; Hassenstab et al., 2010; Segura et al., 2009; Taylor & MacQueen, 2007; Yates et al., 2012). However, many studies rely on individual test scores or clinical cutoffs, which may miss early signs of dysfunction and risk overinterpreting isolated low scores (Palmer et al., 1998). These limitations highlight the value of multivariate, pattern-based methods like latent profile analysis (LPA), which can detect subtle cognitive vulnerabilities. For instance, one study used LPA to demonstrate distinct cognitive subtypes with meaningful biological and demographic differences between older adults with and without Alzheimer’s disease (Scheltens et al., 2016). This research underscores its potential to identify at-risk profiles before clinical impairment emerges and to validate known brain–behavior relationships in clinical populations.

While the link between MetS and cognitive aging is well documented, fewer studies have investigated whether neurobiological changes associated with aging are already detectable in midlife (Kotkowski et al., 2019; Qureshi et al., 2024; Vergoossen et al., 2020). Structural brain age, an MRI-derived biomarker that estimates the biological age of the brain based on anatomical features, has emerged as a sensitive and non-invasive marker of brain aging (Cole et al., 2018). Higher brain predicted age difference (PAD), the discrepancy between estimated brain age and chronological age, has been associated with cognitive decline and increased risk for neurodegenerative disease (Cumplido-Mayoral et al., 2023). In older adults, MetS has been linked to markers of structural brain aging, including cortical thinning and reduced gray matter volume (Shen et al., 2023). However, it remains unclear whether these changes are present earlier in the lifespan and if they are associated with poorer cognitive functioning in those earlier periods.

The current study aimed to offer a more sensitive framework for detecting cognitive and subclinical brain vulnerability in midlife adults with MetS using two complementary tools: (1) LPA to uncover subtle but interpretable patterns in cognitive performance and (2) a biologically grounded, MRI-derived brain-predicted age metric to detect structural brain aging patterns. Specifically, we (1) determined whether cognitive profile differences are detectable among individuals with and without MetS; (2) examined associations between MetS (i.e., group status and individual components) and structural brain age; (3) explored how age within midlife moderates these associations; and (4) examined whether differences in structural brain age are reflected across cognitive profiles.

## Methods

### Participants & inclusion/exclusion criteria

Participants were cognitively unimpaired, community-dwelling adults aged 40 and older without diagnosis of a neurological or psychiatric disorder who previously participated in studies examining metabolic and brain health (Study #1: Neural Consequences of Metabolic Syndrome [*N* = 274]; Study #2: Cognitive Enhancement through Transcranial Laser Therapy [*N* = 196]). Details about study procedures have been previously described (Foret et al., 2021). The two studies were conducted by the same personnel using identical procedures, equipment, and assessments; thus, no harmonization was required (Supplemental Table 1). The present analyses are cross-sectional and used data collected at baseline study visits. Participants were excluded for the following reasons: incomplete demographic data (consented but did not participate in in-person visits; *n* = 94); age above 65 (*n* = 50); incomplete cardiometabolic data (*n* = 37); incomplete cognitive data (*n* = 22); a score less than 24 on the Mini-Mental State Examination (MMSE; *n* = 1) to ensure intact cognitive functioning (Tombaugh & McIntyre, 1992); incomplete neuroimaging data (*n* = 27); and cognitive scores greater or less than 2.5 standard deviations from the sample mean on cognitive measures (*n* = 9) to reduce the influence of extreme outliers on cognitive profile estimation (Dugravot et al., 2015). This resulted in an analytic sample of *N* = 230 (Study #1: *n* = 191; Study #2: 39). Study 1 contributed proportionally more participants with MetS, whereas Study 2 contributed proportionally more participants without MetS. Additional information about participants removed due to incomplete data and results from tests of missingness can be found in Supplemental Table 3. All participants provided written informed consent before enrolling in the study. Study procedures were completed in accordance with the Helsinki Declaration and approved by the Institutional Review Board at the University of Texas at Austin (Study #1: 2011070025; Study #2: 2016090135).

### Cardiovascular health assessment and metabolic syndrome identification

Participants provided self-reported medical history and medication information prior to the health assessment. They were instructed to fast for 12 hours before their appointment to ensure accurate measurement of fasting glucose and lipid levels. Health assessments were scheduled in the morning to standardize timing across participants. Participants attended an in-person health assessment at a university research laboratory. Certified research assistants collected blood samples via venipuncture through the antecubital vein. Fasting glucose levels were measured using standard enzymatic techniques. Serum concentrations of triglycerides, high-density lipoprotein (HDL), low-density lipoprotein (LDL), and total cholesterol were measured immediately following the blood draw using a commercial clinical analyzer. Blood pressure was measured using a manual cuff technique applied to the right brachial arm after participants were seated quietly for at least five minutes. Systolic and diastolic pressures were recorded. Anthropometric measurements were obtained using a balance beam scale. Measurements were taken in the morning after the fasting period, with participants wearing lightweight clothing and no shoes to minimize variability due to daily fluctuations. All assessments were conducted by trained research staff to ensure consistency and reliability across participants.

Based on laboratory health assessment results, self-reported medical history, and current medications, participants were classified according to the presence or absence of five individual metabolic syndrome components: (1) waist circumference( ≥ 102 cm for men, ≥ 88 cm for women); (2) elevated triglycerides (≥ 150 mg/dL), or use of triglyceride-lowering medication; (3) low HDL cholesterol (< 40 mg/dL for men, < 50 mg/dL), or use cholesterol medication; (4) elevated blood pressure (systolic ≥ 130 mmHg or diastolic ≥ 85 mmHg), or use of blood pressure medication; and (5) elevated fasting glucose (≥ 100 mg/dL), or use of insulin-regulating medication. MetS status was assigned according to clinical criteria outlined by Alberti et al. (2009), with participants meeting three or more components classified as having MetS. In addition, the total number of MetS components present was recorded for each participant.

### Neuropsychological assessment and latent profile analysis

**Figure 1.**
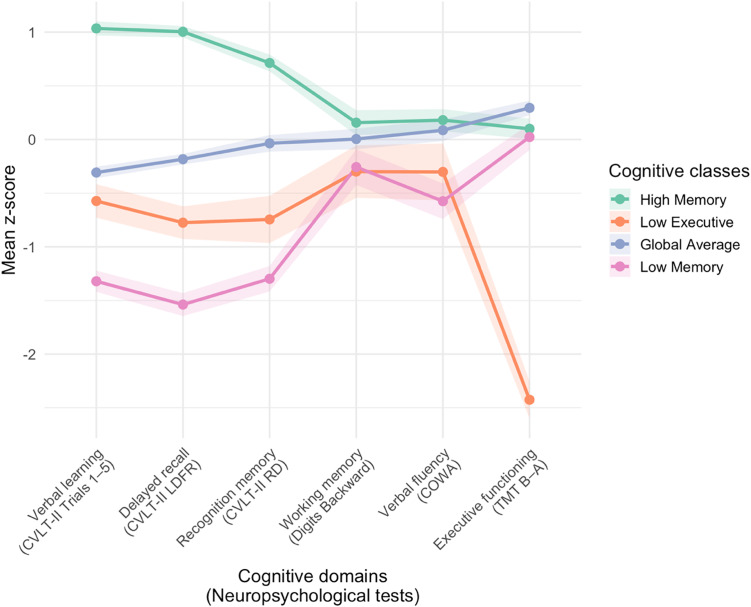
Cognitive classes identified by latent profile analysis. CVLT-II = California Verbal Learning Test – Second Edition; LDFR = long delay free recall; RD = recognition discriminability; COWA = Controlled Oral Word Association; TMT = Trail Making Test. The figure displays the four-class solution identified by latent profile analysis. Each line represents the mean adjusted z-scores for neuropsychological test performance within a cognitive class, with shaded bars indicating standard errors.

Participants completed a battery of neuropsychological tests, as detailed in prior publications (Foret et al., 2021). The present analysis included measures across cognitive domains that have been previously shown to be associated with cardiometabolic health risk in adults (Foret et al., 2021; Lamar et al., 2021): (1) verbal learning (California Verbal Learning Test – Second Edition [CVLT-II] Trials 1–5 Total), (2) delayed recall (CVLT-II Long Delay Free Recall), and (3) recognition memory (CVLT-II Recognition Discriminability) (Delis et al., 2016); (4) working memory (Digit Span Backward) (Wechsler, 2008); (5) verbal fluency (Controlled Oral Word Association [COWA]) (Benton et al., 2017); and (6) executive functioning (Trail Making Test [TMT] Part B time minus Part A time; reverse-scored) (Reitan, 1955). Raw cognitive test scores for the present sample are provided in Supplemental Table 4. Cognitive scores were demographically adjusted for age, sex, and years of education and subsequently z-scored. These sample-specific z-scores were then used as inputs to a LPA conducted using the tidyLPA R package (Rosenberg et al., 2018). The 4-class solution was determined to be the best fit based on AIC, BIC, BLRT, Log Likelihood, and entropy statistics (Supplemental Table 5) and tidyLPA’s analytic hierarchy process (Akogul & Erisoglu, 2017) comparing the 2, 3, 4, and 5-class solutions. The four classes reflected distinct cognitive performance patterns (Supplemental Figure 1, Supplemental Table 6). Figure 1 displays the mean sample-based z-scores for each of the six tasks and across the four profiles. Posterior probabilities for each class (0.89 to 0.95) were determined to be within an acceptable range (Jung & Wickrama, 2008).

Differences in cognitive sample-based z-scores across the 4 LPA-derived cognitive classes were evaluated using Kruskal–Wallis nonparametric tests with Bonferroni adjustment for multiple comparisons. Class 1 demonstrated the strongest performance across memory measures (i.e., verbal learning, delayed recall, recognition memory) compared to the other classes (all *H* > 98.9, *p* < 0.001), we refer to this group as the High Memory profile (*n* = 80). Class 2 demonstrated the poorest performance on the executive functioning task compared to the other classes (*H* = 46.19, *p* < 0.001); this group is labeled the Low Executive profile (*n* = 16). Class 3 scored near the sample mean across all measures without isolated strengths or weaknesses, and is labeled the Global Average profile (*n* = 102). Class 4 demonstrated the poorest performance on memory tasks (i.e., verbal learning, delayed recall) compared to the other classes (all *H* > 160.23, *p* < 0.001); this group is labeled the Low Memory profile (*n* = 32). These cognitive profile labels are intended to provide a concise description of the observed patterns of test performance. Further statistical comparisons of sample and clinical characteristics across the cognitive classes are provided in Supplemental Table 6. Missingness of cognitive data (the indicators used in the LPA) was not significantly predicted by any demographic variable (all *p* > 0.05, Supplemental Table 3.1), consistent with a Missing Completely at Random assumption for the LPA indicators.

### MRI data acquisition and structural brain age

Magnetic resonance imaging (MRI) was conducted on a Siemens 3T Skyra with a 32-channel head coil at the Biomedical Imaging Center (RRID: SCR_021898), a core facility within the Center for Biomedical Research Support at the University of Texas at Austin. High-resolution magnetization prepared rapid gradient echo (MPRAGE) structural images were acquired using the following parameters: 256 × 256 matrix, flip angle = 7°, Field-of-view (FOV) = 24 × 24 cm^2^, 1 mm slice thickness, 0 gap. These T1-weighted (T1w) images were used as inputs to the brainageR software (version 2.1) (Cole, 2019) to predict brain age using a Gaussian Processes regression. This software was trained on data from 7 large-scale neuroimaging studies; additional information can be found at https://github.com/james-cole/brainageR. For each participant, the model-predicted brain age was then used to calculate predicted age difference (PAD), which is the difference between model-predicted age and chronological age. A positive PAD is therefore indicative of a brain that appears older than the chronological age and has been associated with increased risk for dementia (Biondo et al., 2022). Model-predicted age was significantly correlated with chronological age, suggesting prediction accuracy (*r* = 0.693, *p* < 0.001). Because brain age models may underestimate brain age in older populations, we evaluated for age bias and found that PAD was not significantly correlated with chronological age (*r* = −0.117, *p* = 0.078). Nevertheless, age was included as a covariate in all models examining PAD to account for potential age bias (de Lange & Cole, 2020).

### Statistical analyses

All analyses were conducted using R version 4.3.0. Statistical significance was set at *p* < 0.05. Effect sizes and 95% confidence intervals were reported alongside statistical tests to provide estimates of precision and magnitude. Differences in the proportion of participants with MetS across cognitive profile groups were evaluated using a chi-square test of independence. Effect sizes were estimated using Cramér’s V, and pairwise comparisons were conducted with Bonferroni adjustment for multiple comparisons. Analysis of covariance (ANCOVA) was used to examine differences in PAD based on MetS group status and individual metabolic components. Models were adjusted for age and sex, with effect sizes reported as partial eta-squared and adjusted means presented with 95% confidence intervals. To test whether chronological age moderated the association between MetS status and PAD, multiple linear regression was conducted including main effects of age and MetS status, their interaction term (age × MetS), and sex as a covariate. A Johnson–Neyman technique was applied to probe the conditional effect of MetS on PAD across the continuous age variable. This approach identifies the specific range of age for which the association between MetS and PAD is statistically significant, providing corresponding confidence intervals for the estimated simple slopes. This approach provides more granular information by identifying regions of significance within the moderator, which may be obscured in the overall interaction term (Preacher et al., 2006). Finally, differences in PAD across cognitive profile groups were evaluated using a Kruskal–Wallis nonparametric test, due to unequal sample sizes.

## Results

### Sample and clinical characteristics

Demographic and clinical characteristics are presented in Table 1. Seventy-five participants met the clinical criteria for MetS as established by Alberti et al. (2009). There were no significant differences across MetS groups on demographic variables (all *p* > 0.05). There were significant differences in MetS component variables, such that the MetS group displayed a greater presence of each individual MetS component (all *p* < 0.001), as expected. There were significant group differences for some cognitive test scores, such that the MetS group displayed poorer performance on measures of verbal learning and memory (all *p* < 0.05).

**Table 1.** Sample and clinical characteristics

		MetS (*n* = 75)	No MetS (*n* = 155)	Test statistic (t or X^2^)	*p*
Age (mean (SD))		50.29 (6.86)	50.57 (7.00)	0.28	0.779
Sex (*n* (*n*%))	Female	37 (49.33)	96 (61.94)	2.80	0.095
	Male	38 (50.67)	59 (38.06)		
Race/ethnicity (*n* (*n*%))	Asian	2 (2.67)	7 (4.52)	4.69	0.345
	Black	3 (4.00)	12 (7.74)		
	Hispanic	17 (22.67)	27 (17.42)		
	Multiracial	1 (1.33)	0 (0.00)		
	Other	4 (5.33)	6 (3.87)		
	White	47 (62.67)	103 (66.45)		
	N/A	1 (1.33)	0 (0.00)		
Years of education (mean (SD))		16.01 (2.71)	16.59 (2.38)	1.60	0.095
MMSE total score (mean (SD))		28.64 (1.45)	28.72 (1.54)	0.40	0.698
Estimated IQ (mean (SD))					
WASI-II FSIQ-2 ^a^		114.23 (13.70)	110.44 (16.03)	1.66	0.099
WTAR ^b^		110.66 (6.66)	107.00 (10.55)	0.86	0.447
***Metabolic syndrome components***					
MetS components (mean (SD))		3.69 (0.75)	0.94 (0.78)	−25.64	< 0.001
Waist circumference component (*n* (*n*%))	Absent	7 (9.33)	100 (64.52)	59.67	< 0.001
	Present	68 (90.67)	55 (35.48)		
Triglycerides component (*n* (*n*%))	Absent	19 (25.33)	139 (89.68)	94.34	< 0.001
	Present	56 (74.67)	16 (10.32)		
Cholesterol component (*n* (*n*%))	Absent	16 (21.33)	137 (88.39)	99.06	< 0.001
	Present	59 (78.67)	18 (11.61)		
Blood pressure component (*n* (*n*%))	Absent	30 (40.00)	117 (75.48)	26.08	< 0.001
	Present	45 (60.00)	38 (24.52)		
Glucose component (*n* (*n*%))	Absent	26 (34.67)	136 (87.74)	65.85	< 0.001
	Present	49 (65.33)	19 (12.26)		
***Cognitive test performance*** ^***c***^					
CVLT-II Trials 1 – 5 (mean (SD))		−0.27 (0.99)	0.13 (0.98)	2.90	0.004
CVLT-II LDFR (mean (SD))		−0.35 (0.94)	0.17 (0.99)	3.89	< 0.001
CVLT-II Recognition Discriminability (mean (SD))		−0.22 (0.94)	0.10 (1.02)	2.36	0.020
COWA (mean (SD))		−0.10 (1.03)	0.05 (0.99)	1.05	0.296
Digit Span Backward (mean (SD))		−0.03 (1.04)	0.02 (0.98)	0.32	0.747
TMT (mean (SD))		0.04 (1.06)	−0.02 (0.97)	−0.46	0.647

COWA = Controlled Oral Word Association; CVLT-II = California Verbal Learning Test – Second Edition; FSIQ-2 = Full Scale Intelligence Quotient-2; IQ = intelligence quotient; LDFR = Long Delay Free Recall; MetS=metabolic syndrome; MMSE = Mini-Mental Status Examination; SD = standard deviation; TMT = Trail Making Test (Part B minus Part A; reverse-scored); WASI-II = Wechsler Abbreviated Scale of Intelligence—2^nd^ Edition; WTAR = Weschler Test of Adult Reading.

a Study 1 participants only (*n* = 191).

b Study 2 participants only (*n* = 39).

c Sample-specific z-scores adjusted for age, sex, and years of education.

### MetS status differs across cognitive profiles

**Figure 2.**
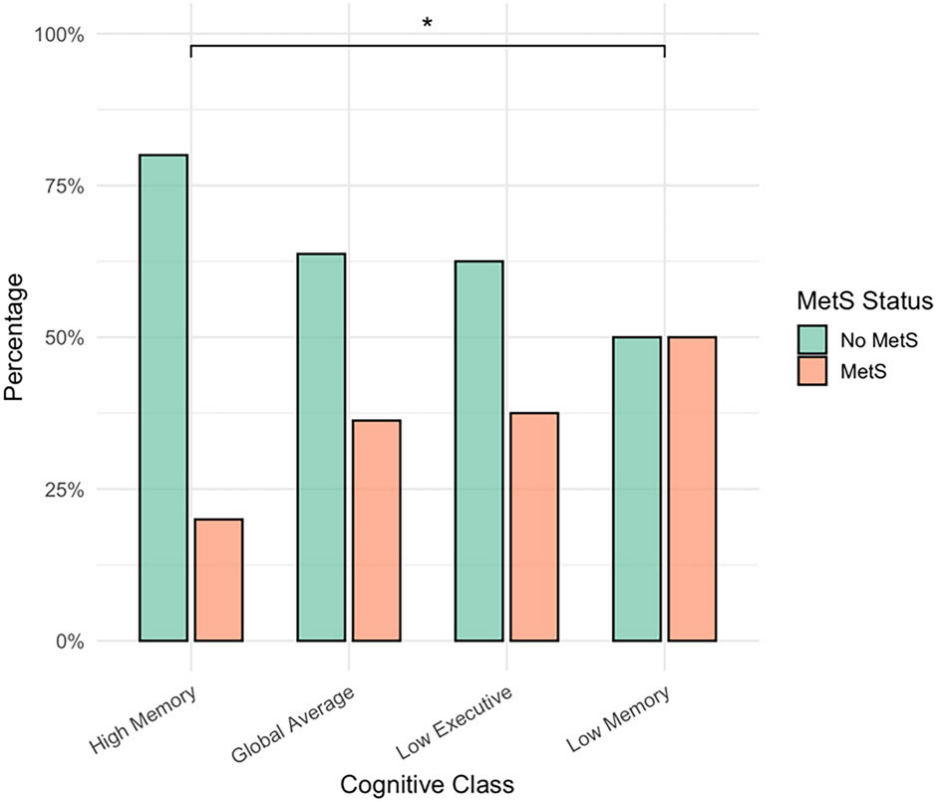
Metabolic Syndrome status differs across cognitive profiles. MetS = Metabolic Syndrome, *Bonferroni-adjusted significance (*p* < 0.05). The figure displays the percentages of participants with and without Metabolic Syndrome (MetS) across the four cognitive classes. The proportion of MetS participants differed significantly across classes (χ^2^ = 10.99, *p* = 0.012, *V* = 0.22). Pairwise comparisons indicated that the proportion of MetS participants was significantly higher in the Low Executive group compared with the High Memory group (*p* = 0.020, Bonferroni-adjusted). The difference between the Global Average and High Memory groups was not significant after Bonferroni correction (*p* = 0.153). No other pairwise comparisons were significant (*p* > 0.05).

There were significant differences in the proportion of MetS participants across the cognitive classes (χ^2^ = 10.99, *p* = 0.012, *V* = 0.22). Pairwise comparisons revealed that, compared with the High Memory group, the proportion of MetS participants was higher in the Low Memory group (*p*-unadjusted = 0.003, p-adjusted = 0.020) and in the Global Average group (p-unadjusted = 0.026), although the latter finding did not survive Bonferroni correction (*p*-adjusted = 0.153). All other pairwise comparisons were not significant (*p* > 0.05) (Figure 2).

### MetS status and elevated triglycerides are associated with structural brain aging

**Figure 3.**
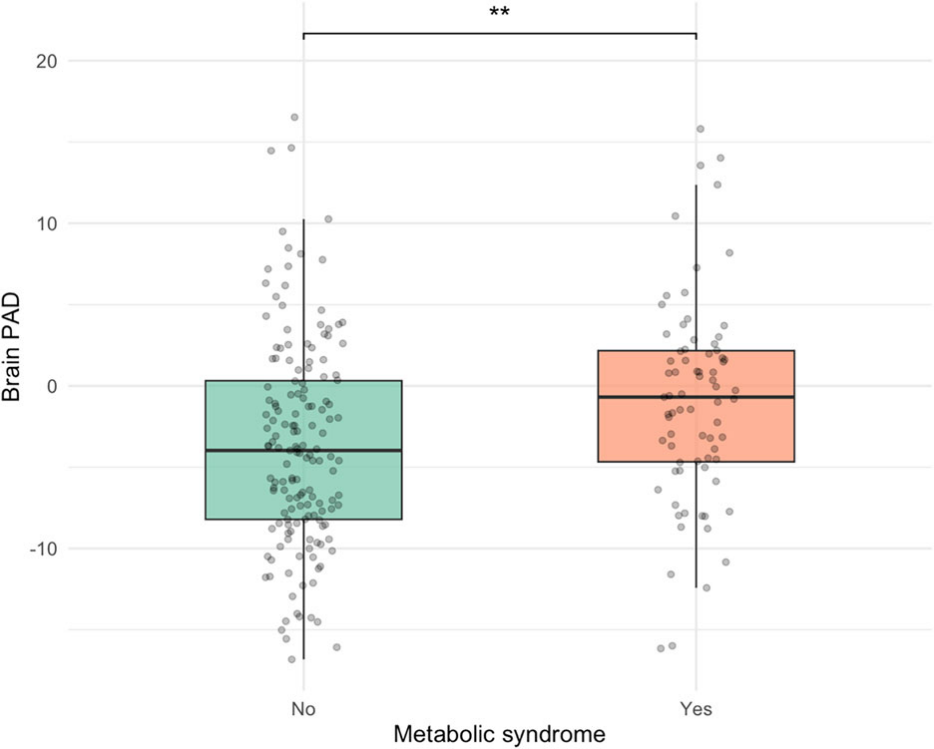
Younger PAD among participants without metS. PAD = predicted age difference, calculated by chronological age minus structural brain age metric; metS = Metabolic syndrome, **p < 0.01. The figure displays box plots illustrating the distribution of brain-PAD among individuals with and without metabolic syndrome. The no metS group exhibited significantly lower brain-PAD, by an average of 2.5 years, compared to the metS group, controlling for age and sex, *F* (1, 226) = 9.16, *p* = .003, partial η^2^ = .03.

Participants without MetS displayed a significantly lower adjusted mean PAD of −3.44 years (SE = 0.52, 95% CI [−4.46, −2.42]) compared to those with MetS, who had an adjusted mean PAD of −1.00 years (SE = 0.73, 95% CI [−2.43, 0.44]). The adjusted mean difference between groups was −2.44 years (SE = 0.89, 95% CI [−4.20, −0.69]), *F* (1, 226) = 9.16, *p* = 0.003, partial η^2^ = 0.03, after covarying for age and sex (Figure 3).

Similarly, participants with the triglyceride component (i.e., elevated triglycerides and/or use of relevant medication) had an adjusted mean PAD of −1.21 years (SD = 6.12), which was 2.28 years higher than those without elevated triglycerides (mean = −3.49, SD = 6.52), 95% CI [0.53, 4.03], *F* (1, 226) = 6.43, *p* = 0.012, partial η^2^ = 0.022. There were no significant differences in PAD across the other individual MetS components (all *p* > 0.05).

### Age moderates the relationship between MetS status and structural brain age

**Figure 4.**
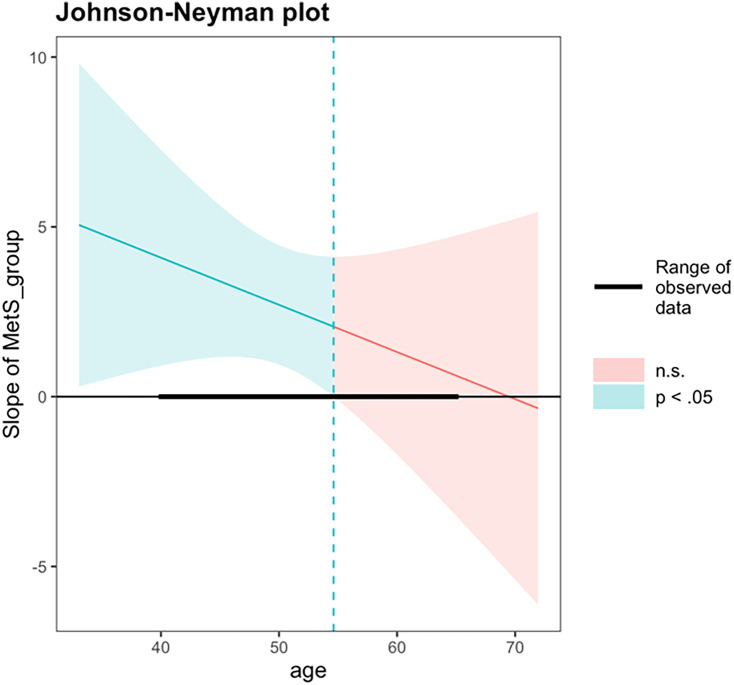
Johnson–Neyman analysis between chronological age and metS status on brain-PAD. PAD = predicted age difference, calculated by chronological age minus structural brain age metric; metS = Metabolic syndrome. A Johnson–Neyman analysis identified a significant region of interaction between ages 40.0 and 54.6, within which metS was significantly associated with higher brain-PAD. Outside of this age range, metS was not associated with brain-PAD.

An exploratory interaction analysis examined whether the association between MetS status and PAD varied as a function of chronological age. The interaction term between MetS status and chronological age did not reach statistical significance (β = −0.14, 95% CI [–0.42, 0.13], *p* = 0.290). The overall model was significant, *F* (4, 225) = 4.30, *p* = 0.002, R^2^ = 0.071. A follow-up Johnson–Neyman analysis was performed to probe whether the association between MetS and PAD differed at specific age ranges. The Johnson–Neyman simple slopes analysis revealed that the association between MetS status and PAD was statistically significant at younger chronological ages but not at older ages. Specifically, at one standard deviation below the mean age (43.5 years), MetS was associated with significantly greater PAD (*b* = 3.60, SE = 1.26, 95% CI [1.13, 6.07], *t* = 2.86, *p* < 0.01). At the mean age (50.5 years), this association remained significant (*b* = 2.64, SE = 0.89, 95% CI [0.90, 4.38], *t* = 2.96, *p* < 0.01). However, at one standard deviation above the mean age (57.4 years), the association was no longer significant (*b* = 1.67, SE = 1.28, 95% CI [−0.86, 4.20], *t* = 1.31, *p* = 0.190). The Johnson–Neyman analysis identified a significant region of interaction between chronological ages 40.0 and 54.6 years, within which adults with MetS exhibited significantly greater PAD compared to those without MetS. Beyond age 54.6 years, differences in PAD by MetS status were no longer statistically significant (Figure 4).

### Structural brain age does not differ across cognitive profiles

A Kruskal–Wallis test indicated no significant difference in PAD across cognitive profile groups (*H*(3) = 0.21, *p* = 0.976) (Supplemental Figure 2).

## Discussion

### Summary

The present findings suggest that the presence of MetS in midlife, compared to no MetS, is associated with relative differences in patterns of cognitive performance and structural brain aging in a cognitively unimpaired sample.

### MetS and LPA-derived cognitive profiles

We employed latent profile analysis to identify distinct cognitive subgroups based on overall cognitive patterns, rather than isolated neuropsychological test scores. Our results revealed that individuals with MetS were more likely to fall into the Low Memory and Global Average cognitive profile groups, whereas those without MetS were more likely to be in the High Memory group. These findings align with prior work that identified similar cognitively defined subgroups in a cognitively unimpaired, older adult sample (Lamar et al., 2021). Importantly, cognitive profiles in their study were also associated with modifiable cardiovascular and lifestyle factors, such as fasting glucose and adherence to a Mediterranean diet. This approach provides a more comprehensive and person-centered understanding of cognitive vulnerability than relying on individual test scores alone. Our findings also suggest that memory may be particularly impacted by the presence of MetS in midlife, which has been similarly reflected in longitudinal studies showing greater worsening of memory performances in those with MetS (Komulainen et al., 2007; Yaffe et al., 2007). The consistency across studies strengthens the case for cognitive subgrouping as a meaningful way to detect early vulnerability, particularly within the domain of memory, and underscores the role of cardiometabolic health in shaping cognitive aging trajectories.

### MetS status and structural brain aging

We identified adults without MetS to have a brain age approximately 2.5 years younger than those with MetS. This is consistent with other studies that have employed brain age models to identify associations between brain age and metabolic risk factors such as elevated body mass index (Kolbeinsson et al., 2020; Shen et al., 2023), elevated blood pressure (Kang et al., 2023; Kolbeinsson et al., 2020; de Lange et al., 2020); diabetes (Franke et al., 2013; Kang et al., 2023; Kolbeinsson et al., 2020), and smoking (Franke et al., 2013). When evaluating individual MetS components within the present study, elevated triglyceride levels – or the use of medication to manage them – was the only component significantly associated with older structural brain age. These results contribute to the growing body of evidence linking midlife metabolic dysfunction with neurodegenerative risk. In particular, elevated triglycerides in midlife have been shown to predict memory decline (Power et al., 2018) and Alzheimer’s disease pathology, specifically amyloid (Aβ) and tau deposition (Nägga et al., 2018). Taken together, this highlights that midlife triglycerides dysregulation may serve a key role in the mechanism of neurobiological aging and subsequent cognitive impacts.

One important finding that the current study adds to the literature is that the association between MetS and structural brain age was most prominent in early midlife and diminished with older age. This pattern suggests that there may be a critical window in which neurobiological vulnerability begins to emerge and early midlife could be a particularly sensitive period for the emergence of brain changes associated with metabolic health. While the overall interaction between MetS and age was not statistically significant across the full age range, follow-up Johnson-–Neyman analysis identified a significant band between ages 40.0 and 54.6 during which MetS was associated with greater PAD. This exploratory approach revealed an effect that may have been masked in the global interaction test, highlighting the importance of probing age-specific effects. These findings suggest that individuals with MetS in early midlife may face heightened neurobiological vulnerability, while differences in PAD by MetS status were no longer significant in later midlife. These findings are consistent with longitudinal evidence showing that the presence of MetS or multimorbidity in midlife – but not later life – is more strongly associated with increased dementia risk over time (Qureshi et al., 2024). While Qureshi and colleagues found that dementia incidence was highest among individuals with MetS in the 60–69 age range, they also emphasized the importance of chronic exposure. Our findings extend this work by showing that brain changes may begin earlier, before clinical symptoms of dementia emerge. Together, these results underscore the importance of considering both the timing and chronicity of metabolic dysfunction when evaluating long-term dementia risk.

### Structural brain aging & cognitive performance

Although MetS was independently associated with differences in both cognitive profiles and brain aging, we did not find a direct relationship between structural brain age and cognitive profiles. Given that our brain age metric captures global age-related changes rather than region-specific aging (e.g., hippocampal atrophy), it is possible that the subtle cognitive dysfunction observed within our sample is driven by more precise changes to neural structures. Future research should continue to examine neurobiological measures of brain aging (e.g., regional brain volumes, functional reorganization, neuroinflammatory markers) that may underlie the relationship between cardiovascular risk and poorer cognitive performance in midlife.

### Strengths & limitations

This study’’s strengths include comprehensive neuropsychological and cardiovascular evaluations, as well as the use of novel imaging and analytic techniques to detect subtle vulnerabilities in midlife. In addition, approximately one-fifth of the sample identified as Hispanic, providing representation of a group often underrepresented in neuroimaging research and modestly enhancing the diversity and relevance of the findings.

To address potential cohort differences, we replicated all analyses in Study 1 alone, which had a higher prevalence of MetS relative to Study 2. Results reveal comparable effect sizes and patterns to the full sample, supporting that the observed associations between MetS and cognitive/brain health are not driven by sampling differences (see Supplemental Material for further details). Additionally, a proportion of participants who initially consented had incomplete data due to attrition and logistical factors (e.g., scheduling constraints, visit burden). Logistic regression models further revealed that older age was associated with a greater likelihood of missing cardiovascular data, and male sex was associated with a greater likelihood of missing neuroimaging data. Importantly, age and sex were included as covariates in all primary analyses and account for these patterns of missingness.

Although participants with MetS exhibited relatively older-appearing brains compared to their peers without MetS, the overall predicted brain age in our sample remained lower than chronological age. Several factors may account for this unexpected pattern. First, our sample was relatively high in educational attainment, which is a well-established protective factor associated with cognitive reserve and brain health (Clouston et al., 2020). Second, the brain age model was trained on a large and more demographically and clinically diverse sample across 7 sites worldwide, which may contribute to systematic differences when applied to our regionally specific cohort recruited within a university setting. Together, these factors likely attenuated the absolute values of predicted brain age while still preserving within-sample differences associated with MetS.

This study’’s cross-sectional design limits the ability to determine temporal precedence, elucidate the mechanisms underlying the observed associations, and ensure that cohort effects (e.g., participation of older adults without cognitive impairment, who may have been healthier in early middle age) do not contribute to the present findings.

## Conclusions

This study offers a framework for detecting cognitive and subclinical brain vulnerability in midlife adults with MetS using latent profile analysis and brain age prediction models. The results reveal that subtle cognitive and brain vulnerabilities associated with MetS, relative to no MetS, can be detected in midlife, with the greatest associations evident in early midlife. Identifying modifiable health behaviors – such as diet, physical activity, sleep, and social engagement – that may moderate these associations could help determine who is most at risk for MetS-related cognitive decline and who may benefit most from early intervention. These findings underscore the importance of targeting cardiometabolic health during early midlife to reduce the risk of cognitive impairment and dementia in later life.

## Supporting information

Gallagher et al. supplementary material Gallagher et al. supplementary material

## Supplementary material

The supplementary material for this article can be found at https://doi.org/10.1017/S1355617725101604.
