# Supplementary material for: Hidden risk: Latent cognitive profiles and structural brain age reveal vulnerability in midlife metabolic syndrome
Source: J Int Neuropsychol Soc. 2025 Nov 24;31(9-10):627–35. doi: 10.1017/S1355617725101604 (PMC13183333; doi:10.1017/S1355617725101604)
Supplement: Gallagher et al. supplementary material [file S1355617725101604sup001.docx]

**Supplemental Table 1: Study Procedures**

| **Study Procedure** | **Study 1** | **Study 2** |
| --- | --- | --- |
| Recruitment | Flyers (UT Austin campus, grocery stores, bus stops), bus ads, Craigslist, Austin American-Statesman newspaper ads | Newspaper and Craigslist ads; flyers across greater Austin, TX |
| Inclusion Criteria | Adults 40–60, with and without cardiometabolic risk factors | Age ≥45; women postmenopausal; CDR = 0 or 0.5 (MCI permitted if no major functional impairment) |
| Exclusion Criteria | Neurological disease (stroke, seizure, Parkinson’s, TBI, MS, meningitis), major psychiatric illness (schizophrenia, bipolar), substance abuse diagnosis/hospitalization, MRI contraindication, FSIQ <80 | Neurological disorders (stroke, seizure, Parkinson’s, TBI, MS, meningitis); major psychiatric illness (schizophrenia, bipolar); psychosis or violent behavior; pregnancy; MRI contraindications (including BMI >40); vascular abnormalities on MRI |
| Cognitive Measures | MMSE, WASI-II (Matrix Reasoning, Vocabulary), Stroop Color-Word, COWA, Trail Making Test A & B, WAIS-IV Digit Span, CVLT-II | MMSE, Dementia Rating Scale, Clinical Dementia Rating, WTAR, CVLT-II, BVMT-R, Trail Making Test A & B, WAIS Digit Span, COWA, Stroop Color-Word, Boston Naming Test |
| Health Assessment | Venipuncture 12-hour fasting blood draw (lipids, glucose, insulin); standard enzymatic method for blood analyses (Alere Cholestech LDX); resting blood pressure (Omron VP-200); weight, height, waist/hip circumference via tape measure and balance beam scale | Venipuncture 12-hour fasting blood draw (lipids, glucose, insulin, inflammatory proteins); standard enzymatic method for blood analyses (Alere Cholestech LDX); resting blood pressure (Omron VP-200); weight, height, waist/hip circumference via tape measure and balane beam scale; DEXA body composition; arterial stiffness (ultrasound); saliva sample (ApoE genotyping) |
| MRI Scanner | Siemens Skyra 3T | Siemens Skyra 3T |

**Supplemental Table 2: MRI Acquistion**

| **MRI Parameter** | **Study 1** | **Study 2** |
| --- | --- | --- |
| Coil | 32-channel head coil (HEA; HEP) | 32-channel head coil (HEA; HEP) |
| Acquisition Type | 3D | 3D |
| Sequence Name | t1_mprage_sag_p2_iso_1_Schnyer (*tfl3d1_16ns) | t1_mprage_sag_p2_iso_1_Schnyer (*tfl3d1_16ns) |
| Orientation | Sagittal | Sagittal |
| Scanning Sequence | GR/IR | GR/IR |
| Sequence Variant | SK/SP/MP | SK/SP/MP |
| Scan Options | IR | IR |
| TR | 2530 ms | 2530 ms |
| TE | 3.37 ms | 3.37 ms |
| TI | 1100 ms | 1100 ms |
| Flip Angle | 7° | 7° |
| Base Resolution | 256 | 256 |
| Acquisition Matrix | 256 × 256 | 256 × 256 |
| Slice Thickness | 1 mm | 1 mm |
| FOV | 24 × 24 cm² | 24 × 24 cm² |
| Phase Encoding Direction | ROW | ROW |
| Parallel Imaging | GRAPPA, factor 2 | GRAPPA, factor 2 |
| Partial Fourier | 1 | 1 |
| Phase Resolution | 1 | 1 |
| Pixel Bandwidth | 200 Hz/pixel | 200 Hz/pixel |
| Dwell Time | 9.8 μs | 9.8 μs |
| Nonlinear Gradient Correction | No | No |
| Spoiling | Yes | Yes |
| Patient Position | Head First Supine (HFS) | Head First Supine (HFS) |
| Software Version | syngo MR D11 | syngo MR E11 |
| Institution | Univ of Texas at Austin IRC | UT Austin IRC |
| Procedure Description | Haley^MetS | Haley^LLLT |

**Replication of main results and comparisons of effect sizes using only Study 1 (n=191):**

Given the differences in the prevalence of MetS between Study 1 (40%) and Study 2 (10%), we repeated all primary analyses in Study 1 alone (n = 191). Results were consistent with those from the full sample, as described below.

*Full sample: There were significant differences in the proportion of MetS participants across the cognitive classes (χ²=10.99, p=0.012, V=0.22).*

Study 1 only: Similarly, there were significant differences in the proportion of MetS participants across the cognitive classes (χ²=8.27, p=0.041, V=0.21).

*Full sample:* *The adjusted mean difference in PAD between MetS groups was -2.44 years (SE=0.89, 95% CI [-4.20, -0.69]), F(1, 226)=9.16, p=0.003, partial η²=0.03, after covarying for age and sex.*

Study 1 only: PAD remained significantly different between MetS groups, after covarying for age and sex, F(1, 187)=7.78, p=0.006, partial η²=0.04.

*Full sample: Participants with the triglyceride component (i.e., elevated triglycerides and/or use of relevant medication) had an adjusted mean PAD of -1.21 years (SD=6.12), which was 2.28 years higher than those without elevated triglycerides (mean=-3.49, SD=6.52), 95% CI [0.53, 4.03], F(1, 226)=6.43, p=0.012, partial η²=0.022.*

Study 1 only: PAD remained significantly different between participants with and without the triglyceride component, after covarying for age and sex, F(1, 187)=5.14, p=0.025, partial η²=0.025.

*Full sample:* *The interaction term between MetS status and chronological age did not reach statistical significance (β=-0.14, 95% CI [–0.42, 0.13], p=0.290). The overall model was significant, F(4, 225)=4.30, p=0.002, R²=0.071.*

Study 1 only: Similarly, the interaction term between MetS status and chronological age did not reach statistical significance (β=-0.15, 95% CI [–0.29, 0.00], p=0.311). The overall model remained significant, F(4, 186)=3.66, p=0.007, R²=0.073.

*Full sample: Johnson-Neyman analysis simple slopes analysis revealed at one standard deviation below the mean age (43.5 years), MetS was associated with significantly greater PAD (b=3.60, SE=1.26, 95% CI [1.13, 6.07], t=2.86, p < 0.01). At the mean age (50.5 years), this association remained significant (b=2.64, SE=0.89, 95% CI [0.90, 4.38], t=2.96, p < 0.01). However, at one standard deviation above the mean age (57.4 years), the association was no longer significant (b=1.67, SE=1.28, 95% CI [–0.86, 4.20], t=1.31, p=0.190). The Johnson-Neyman analysis identified a significant region of interaction between chronological ages 40.0 and 54.6 years, within which adults with MetS exhibited significantly greater PAD compared to those without MetS. Beyond age 54.6 years, differences in PAD by MetS status were no longer statistically significant (Figure 3).*

Study 1 only: At one standard deviation below the mean age (42.6 years), MetS was associated with significantly greater PAD (b=3.82, SE=1.37, 95% CI [1.13, 6.52], t=2.80, p < 0.01). At the mean age (49.1 years), this association remained significant (b=2.85, SE=0.96, 95% CI [0.97, 4.74], t=2.99, p < 0.01). However, at one standard deviation above the mean age (55.5 years), the association was no longer significant (b=1.88, SE=1.31, 95% CI [–0.71, 4.47], t=1.31, p=0.15). The Johnson-Neyman analysis identified a significant region of interaction between chronological ages 40.0 and 53.3 years, within which adults with MetS exhibited significantly greater PAD compared to those without MetS.

**Missingness:**

As described in the main text, participants were excluded for the following reasons: incomplete demographic data (consented but did not participate in in-person visits) (n=94); age above 65 (n=50); incomplete cardiometabolic data (n=37); incomplete cognitive data (n=22); a score less than 24 on the Mini-Mental State Examination (MMSE; n=1); incomplete neuroimaging data (n=27); and cognitive scores greater or less than 2.5 standard deviations from the sample mean on cognitive measures (n=9). This resulted in an analytic sample of N=230 (Study #1: n=191; Study #2: 39).

Among those excluded due to missing cardiometabolic data (n=37), n=21 (56.76%) were missing data for 1 metabolic syndrome (MetS) component, n=5 (13.51%) were missing data for 2 MetS components, n=5 (13.51%) were missing data for 3 MetS components, n=1 (2.70%) was missing data for 4 MetS components, and n=5 (13.51%) were missing data for all 5 MetS components. Among those excluded due to missing cardiometabolic data (n=37), n=17 (45.95%) were missing waist circumference data, n=20 (54.05%) were missing triglyceride component data, n=14 (37.84%) were missing cholesterol component data, n=11 (29.73%) were missing blood pressure component data, and n=13 (35.14%) were missing glucose component data.

To test MAR, we conducted logistic regressions to predict missingness of cardiometabolic, cognitive, and neuroimaging data using age, sex, education, and race/ethnicity. Results are displayed in Supplemental Table 3.1 below. Statistical comparisons of sample characteristics between the analytic sample and participants excluded due to incomplete data are provided below in Supplemental Table 3.2.

**Supplemental Table 3.1:** **Results of logistic regression analyses to test missingness at random**

| **Dependent variable** |  | **OR** | **95% CI** | **p** |
| --- | --- | --- | --- | --- |
| Missing cardiometabolic data | Age | 1.06 | [1.00, 1.11] | 0.03 |
|  | Sex | 1.05 | [0.51, 2.14] | 0.89 |
|  | Education | 0.96 | [0.83, 1.11] | 0.60 |
|  | Race/ethnicity | 1.09 | [0.81, 1.43] | 0.53 |
| Missing cognitive data | Age | 1.00 | [0.94, 1.06] | 0.96 |
|  | Sex | 0.66 | [0.24, 1.63] | 0.38 |
|  | Education | 0.92 | [0.77, 1.10] | 0.40 |
|  | Race/ethnicity | 1.13 | [0.79, 1.56] | 0.47 |
| Missing neuroimaging data | Age | 1.04 | [0.98, 1.11] | 0.20 |
|  | Sex | 6.37 | [2.46, 19.9] | <0.001 |
|  | Education | 0.88 | [0.74, 1.04] | 0.14 |
|  | Race/ethnicity | 1.17 | [0.86, 1.56] | 0.30 |

**Supplemental Table 3.2:** **Sample characteristics of participants excluded due to missing data**

|  |  | **Analytic Sample (N=230)** | **Excluded due to missing cardiometabolic data (n=37)** | **Test statistic**  **(t or X^2^)** | **p** |
| --- | --- | --- | --- | --- | --- |
| Age (mean (SD)) | | 50.48 (6.94) | 53.00 (7.83) | 1.85 | 0.071 |
| Sex (n (n%)) | Female | 133 (57.83) | 21 (56.76) | 0.00 | 1.000 |
|  | Male | 97 (42.17) | 16 (43.24) |  |  |
| Race/ethnicity (n (n%)) | Asian | 9 (3.91) | 1 (2.70) | 3.40 | 0.639 |
|  | Black | 15 (6.52) | 4 (10.81) |  |  |
|  | Hispanic | 44 (19.13) | 6 (16.22) |  |  |
|  | Multiracial | 1 (0.43) | 1 (2.70) |  |  |
|  | Other | 10 (4.35) | 2 (5.41) |  |  |
|  | White | 150 (65.22) | 23 (62.16) |  |  |
|  | N/A | 1 (0.43) | 0 (0.00) |  |  |
| Years of education (mean (SD)) | | 16.40 (2.50) | 16.22 (2.31) | -0.45 | 0.655 |
| MMSE total score (mean (SD)) | | 28.70 (1.51) | 28.21 (1.95) | -1.40 | 0.169 |
| Estimated IQ (mean (SD)) | |  |  |  |  |
| WASI-II FSIQ-2 ^a^ | | 113.50 (11.90) | 110.44 (16.03) | 0.23 | 0.819 |
| WTAR ^b^ | | 111.20 (7.11) | 108.50 (9.70) | -1.04 | 0.312 |

|  |  | **Analytic Sample (N=230)** | **Excluded due to missing cognitive data (n=22)** | **Test statistic**  **(t or X^2^)** | **p** |
| --- | --- | --- | --- | --- | --- |
| Age (mean (SD)) | | 50.48 (6.94) | 50.14 (7.61) | -0.20 | 0.841 |
| Sex (n (n%)) | Female | 133 (57.83) | 15 (68.18) | 0.51 | 0.474 |
|  | Male | 97 (42.17) | 7 (31.82) |  |  |
| Race/ethnicity (n (n%)) | Asian | 9 (3.91) | 0 (0.00) | 24.25 | <0.001 |
|  | Black | 15 (6.52) | 1 (4.55) |  |  |
|  | Hispanic | 44 (19.13) | 3 (13.64) |  |  |
|  | Multiracial | 1 (0.43) | 3 (13.64) |  |  |
|  | Other | 10 (4.35) | 0 (0.00) |  |  |
|  | White | 150 (65.22) | 15 (68.18) |  |  |
|  | N/A | 1 (0.43) | 0 (0.00) |  |  |
| Years of education (mean (SD)) | | 16.40 (2.50) | 15.82 (2.22) | -1.17 | 0.254 |

|  |  | **Analytic Sample (N=230)** | **Excluded due to missing imaging (n=27)** | **Test statistic**  **(t or X^2^)** | **p** |
| --- | --- | --- | --- | --- | --- |
| Age (mean (SD)) | | 50.48 (6.94) | 52.11 (6.22) | 1.27 | 0.211 |
| Sex (n (n%)) | Female | 133 (57.83) | 5 (18.52) | 13.48 | <0.001 |
|  | Male | 97 (42.17) | 22 (81.48) |  |  |
| Race/ethnicity (n (n%)) | Asian | 9 (3.91) | 3 (1.11) | 7.14 | 0.210 |
|  | Black | 15 (6.52) | 1 (3.70) |  |  |
|  | Hispanic | 44 (19.13) | 4 (14.81) |  |  |
|  | Multiracial | 1 (0.43) | 1 (3.70) |  |  |
|  | Other | 10 (4.35) | 2 (7.41) |  |  |
|  | White | 150 (65.22) | 16 (59.26) |  |  |
|  | N/A | 1 (0.43) | 0 (0.00) |  |  |
| Years of education (mean (SD)) | | 16.40 (2.50) | 15.93 (2.46) | -0.95 | 0.350 |
| MMSE total score (mean (SD)) | | 28.70 (1.51) | 28.56 (1.55) | -0.44 | 0.659 |
| Estimated IQ (mean (SD)) | |  |  |  |  |
| WASI-II FSIQ-2 ^a^ | | 112.80 (14.68) | 109.60 (13.18) | -1.13 | 0.266 |
| WTAR ^b^ | | 111.20 (7.11) | 93.50 (7.78) | -3.15 | 0.180 |

FSIQ-2=Full Scale Intelligence Quotient-2; IQ=intelligence quotient; MMSE=Mini-Mental Status Examination; SD=standard deviation; WASI-II=Wechsler Abbreviated Scale of Intelligence—2^nd^ Edition; WTAR=Weschler Test of Adult Reading. Of the 37 participants missing cardiometabolic data, n=3 were missing cognitive data (i.e., MMSE, Estimated IQ).

^a^ Study 1 participants only (Analytic Sample n=191)

^b^ Study 2 participants only (Analytic Sample n=39)

**Supplemental Table 4: Raw neuropsychological test scores for full sample (N=230)**

| **Test** | **Mean (SD)** |
| --- | --- |
| CVLT Trials 1-5 Total | 52.22 (9.58) |
| CVLT Long Delay Free Recall | 11.75 (2.68) |
| CVLT Recognition Discriminability | 3.04 (0.70) |
| COWA Total | 42.57 (10.82) |
| Digit Span Backward | 9.17 (2.33) |
| Trails B (in seconds) | 61.93 (21.36) |

COWA = Controlled Oral Word Association; CVLT = California Verbal Learning Test; SD = standard deviation.

**Supplemental Table 5: LPA fit indices for 2-5 class solutions**

|  |  | AIC | BIC | Log Likelihood | Entropy | Prob (min, max) | BLRT | Sample for each identified class |
| --- | --- | --- | --- | --- | --- | --- | --- | --- |
| 2 Classes |  | 3717.55 | 3782.87 | -1839.77 | 0.77 | 0.93, 0.94 | 0.010 | 1 = 131 (56.96%)  2 = 99 (43.04%) |
| 3 Classes |  | 3691.16 | 3780.55 | -1819.58 | 0.82 | 0.90, 0.94 | 0.010 | 1 = 117 (50.87%)  2 = 16 (6.96%)  3 = 97 (42.17%) |
| **4 Classes** |  | **3611.35** | **3724.80** | **-1772.67** | **0.85** | **0.89, 0.95** | **0.010** | **1 = 80 (34.78%)**  **2 = 16 (6.96%)**  **3 = 102 (44.35%)**  **4 = 32 (13.91%)** |
| 5 Classes |  | 3612.81 | 3750.34 | -1766.41 | 0.80 | 0.76, 0.92 | 0.297 | 1 = 55 (23.91%)  2 = 16 (6.96%)  3 = 103 (44.78%)  4 = 24 (10.43%)  5 = 32 (13.91%) |

AIC = Akaike’s Information Criterion; BIC = Bayesian Information Criterion; BLRT = Bootstrapped Likelihood Ratio Test.

**Supplemental Table 6: Sample and clinical characteristics of LPA 4-class solution**

|  |  | **Class 1:**  **High**  **Memory (n=80)** | **Class 2:**  **Low Executive (n=16)** | **Class 3: Global Average (n=102)** | **Class 4:**  **Low**  **Memory (n=32)** | **Test statistic**  **(H or X^2^)** | **p** | **Pairwise comparisons** |
| --- | --- | --- | --- | --- | --- | --- | --- | --- |
| Age (mean (SD)) | | 50.56 (6.88) | 50.63 (7.12) | 50.09 (7.21) | 50.44 (6.22) | 1.61 | 0.657 | — |
| Sex (n (n%)) | Female | 46 (57.50) | 8 (50.00) | 59 (57.84) | 20 (62.50) | 0.69 | 0.875 | — |
|  | Male | 34 (42.50) | 8 (50.00) | 43 (42.16) | 12 (37.50) |  |  |  |
| Race/ethnicity (n (n%)) | Asian | 1 (1.25) | 0 (0.00) | 4 (3.92) | 4 (12.50) | 23.59 | 0.072 | — |
|  | Black | 3 (3.75) | 0 (0.00) | 9 (8.82) | 3 (9.38) |  |  |  |
|  | Hispanic | 14 (17.50) | 5 (31.25) | 20 (19.61) | 5 (15.62) |  |  |  |
|  | Multiracial | 1 (1.25) | 0 (0.00) | 0 (0.00) | 0 (0.00) |  |  |  |
|  | Other | 1 (1.25) | 2 (12.50) | 4 (3.92) | 3 (9.38) |  |  |  |
|  | White | 60 (75.00) | 8 (50.00) | 65 (63.73) | 17 (53.12) |  |  |  |
|  | N/A | 0 (0.00) | 1 (6.25) | 0 (0.00) | 0 (0.00) |  |  |  |
| Years of education (mean (SD)) | | 16.28 (2.47) | 15.44 (1.86) | 16.55 (2.39) | 16.73 (3.10) | 4.84 | 0.184 | — |
| MMSE total score (mean (SD)) | | 28.75 (1.53) | 27.88 (1.36) | 28.89 (1.40) | 28.34 (1.73) | 11.40 | 0.010 | 1, 3 > 2 |
| Estimated IQ (mean (SD)) | |  |  |  |  |  |  |  |
| WASI-II FSIQ-2 ^a^ | | 116.55 (14.55) | 105.25 (12.49) | 113.03 (14.33) | 106.86 (14.49) | 10.56 | 0.014 | 1 > 4 |
| WTAR ^b^ | | 111.81 (6.31) | 105.25 (11.32) | 113.00 (5.83) | 107.75 (8.58) | 3.48 | 0.323 | — |
| ***Metabolic Syndrome*** | | | | | | | |  |
| MetS group (n (n%)) | No MetS  MetS | 64 (80.00)  16 (20.00) | 10 (62.50)  6 (37.50) | 65 (63.73)  37 (36.27) | 16 (50.00)  16 (50.00) | 10.99 | 0.012 | 1 > 4 |
| MetS components (mean (SD)) |  | 1.41 (1.45) | 2.06 (1.77) | 2.04 (1.41) | 2.16 (1.61) | 11.31 | 0.010 | 3 > 1 |
| Waist circumference component (n (n%)) | Absent | 46 (57.50) | 8 (50.00) | 41 (40.20) | 12 (37.50) | 6.64 | 0.084 | — |
|  | Present | 34 (42.50) | 8 (50.00) | 61 (59.80) | 20 (62.50) |  |  |  |
| Triglycerides component (n (n%)) | Absent | 59 (73.75) | 10 (62.50) | 69 (67.65) | 20 (62.50) | 1.86 | 0.602 | — |
|  | Present | 21 (26.25) | 6 (37.50) | 33 (32.35) | 12 (37.50) |  |  |  |
| Cholesterol component (n (n%)) | Absent | 62 (77.50) | 10 (62.50) | 64 (62.75) | 17 (53.12) | 7.68 | 0.053 | — |
|  | Present | 18 (22.50) | 6 (37.50) | 38 (37.25) | 15 (46.88) |  |  |  |
| Blood pressure component (n (n%)) | Absent | 57 (71.25) | 9 (56.25) | 62 (60.78) | 19 (59.38) | 2.99 | 0.393 | — |
|  | Present | 23 (28.75) | 7 (43.75) | 40 (39.22) | 13 (40.62) |  |  |  |
| Glucose component (n (n%)) | Absent | 63 (78.75) | 10 (62.50) | 66 (64.71) | 23 (71.88) | 4.78 | 0.189 | — |
|  | Present | 17 (21.25) | 6 (37.50) | 36 (35.29) | 9 (28.12) |  |  |  |
| ***Cognitive Test Performance ^c^*** | | | | | |  |  |  |
| CVLT-II Trials 1-5  (mean (SD)) |  | 1.04 (0.59) | -0.57 (0.63) | -0.31 (0.54) | -1.32 (0.56) | 160.23 | <0.001 | 1 > 2, 3, 4  2, 3 > 4 |
| CVLT-II LDFR (mean (SD)) |  | 1.00 (0.48) | -0.77 (0.61) | -0.18 (0.50) | -1.54 (0.60) | 169.79 | <0.001 | 1 > 2, 3, 4  2, 3 > 4  3 > 2 |
| CVLT-II Recognition Discriminability (mean (SD)) |  | 0.71 (0.70) | -0.74 (0.88) | -0.04 (0.78) | -1.30 (0.67) | 98.95 | <0.001 | 1 > 2, 3, 4  3 > 2, 4 |
| COWA (mean (SD)) |  | 0.18 (0.92) | -0.30 (1.06) | 0.09 (1.01) | -0.57 (0.94) | 15.72 | 0.001 | 1, 3 > 4 |
| Digit Span Backward (mean (SD)) |  | 1.16 (1.05) | -0.30 (0.98) | 0.00 (0.97) | -0.26 (0.95) | 6.47 | 0.091 | — |
| TMT (mean (SD)) |  | 0.10 (0.84) | -2.42 (0.69) | 0.29 (0.68) | 0.02 (0.68) | 46.19 | <0.001 | 1, 3, 4 > 2 |

COWA=Controlled Oral Word Association; CVLT-II=California Verbal Learning Test – Second Edition; FSIQ-2=Full Scale Intelligence Quotient-2; IQ=intelligence quotient; LDFR=Long Delay Free Recall; MetS=metabolic syndrome; MMSE=Mini-Mental Status Examination; SD=standard deviation; TMT=Trail Making Test (Part B minus Part A; reverse-scored); WASI-II=Wechsler Abbreviated Scale of Intelligence—2^nd^ Edition; WTAR=Weschler Test of Adult Reading.

^a^ Study 1 participants only (n=191)

^b^ Study 2 participants only (n=39)

^c^ Sample-specific z-scores adjusted for age, sex, and years of education

Differences in sample and clinical characteristics across the four cognitive profile groups were evaluated using Kruskal-Wallis tests (given unequal sample sizes) and chi-square of tests independence, with Bonferroni adjustment for multiple comparisons. Sample characteristics (age, sex, race/ethnicity, years of education) did not differ across the cognitive classes (p’s>0.05). MMSE total score significantly differed across the groups (H(3)=11.40, p=0.010), such that the High Memory (adjusted p=0.038) and Global Average (adjusted p=0.009) groups displayed higher MMSE scores relative to the Low Executive group. WASI-II FSIQ-2 significantly differed across the groups (H(3)=10.56, p=0.014), such that the High Memory group displayed a higher mean estimated IQ relative to the Low Memory group (adjusted p=0.048); WTAR estimated IQ did not significantly differ across the groups (p=0.323). There were significant differences in the proportion of MetS participants across the cognitive classes (χ²=10.99, p=0.012), such that the High Memory group displayed a higher proportion of No MetS participants relative to the Low Memory group (adjusted p=0.020). The mean number of MetS components also differed across the groups (H(3)=11.31, p=0.010), such that the Global Average group displayed a higher mean number of MetS components relative to the High Memory group (adjusted p=0.008).

As expected, there were differences in mean sample-specific z-scores (adjusted for age, sex, and years of education) across the four cognitive classes. CVLT-II Trials 1-5 performance significantly differed across the groups (H(3)=160.23, p<0.001), such that the High Memory group displayed a higher mean score relative to the other 3 cognitive groups (adjusted p’s<0.001), and the Low Memory group displayed a lower mean score relative to the other 3 groups (adjusted p’s<=0.002). Similarly, CVLT-II LDFR performance significantly differed across the groups (H(3)=169.79, p<0.001), such that the High Memory group displayed a higher mean score relative to the other 3 cognitive groups (adjusted p’s<0.001), the Low Memory group displayed a lower mean score relative to the other 3 groups (adjusted p’s<0.001), and the Global Average group displayed a higher mean score relative to the Low Executive group (adjusted p=0.001). CVLT-II Recognition Discriminability performance significantly differed across the groups (H(3)=98.95, p<0.001), such that the High Memory group displayed a higher mean score relative to the other 3 cognitive groups (adjusted p’s<0.001), and the Global Average groups displayed a higher mean score relative to the Low Executive (adjusted p=0.015) and Low Memory (adjusted p<0.001) groups. COWA performance significantly differed across the groups (H(3)=15.72, p=0.001), such that the Low Memory group displayed a lower mean score relative to the High Memory (adjusted p=0.001) and Global Average (adjusted p=0.012) groups. TMT performance significantly differed across the groups (H(3)=46.19, p<0.001), such that the Low Executive group displayed a lower mean score relative to the other 3 cognitive groups (adjusted p’s<0.001). Digit Span Backward did not significantly differ across the cognitive classes (p=0.091).

**Supplemental Figure 1: 4-class solution identified by latent profile analysis**


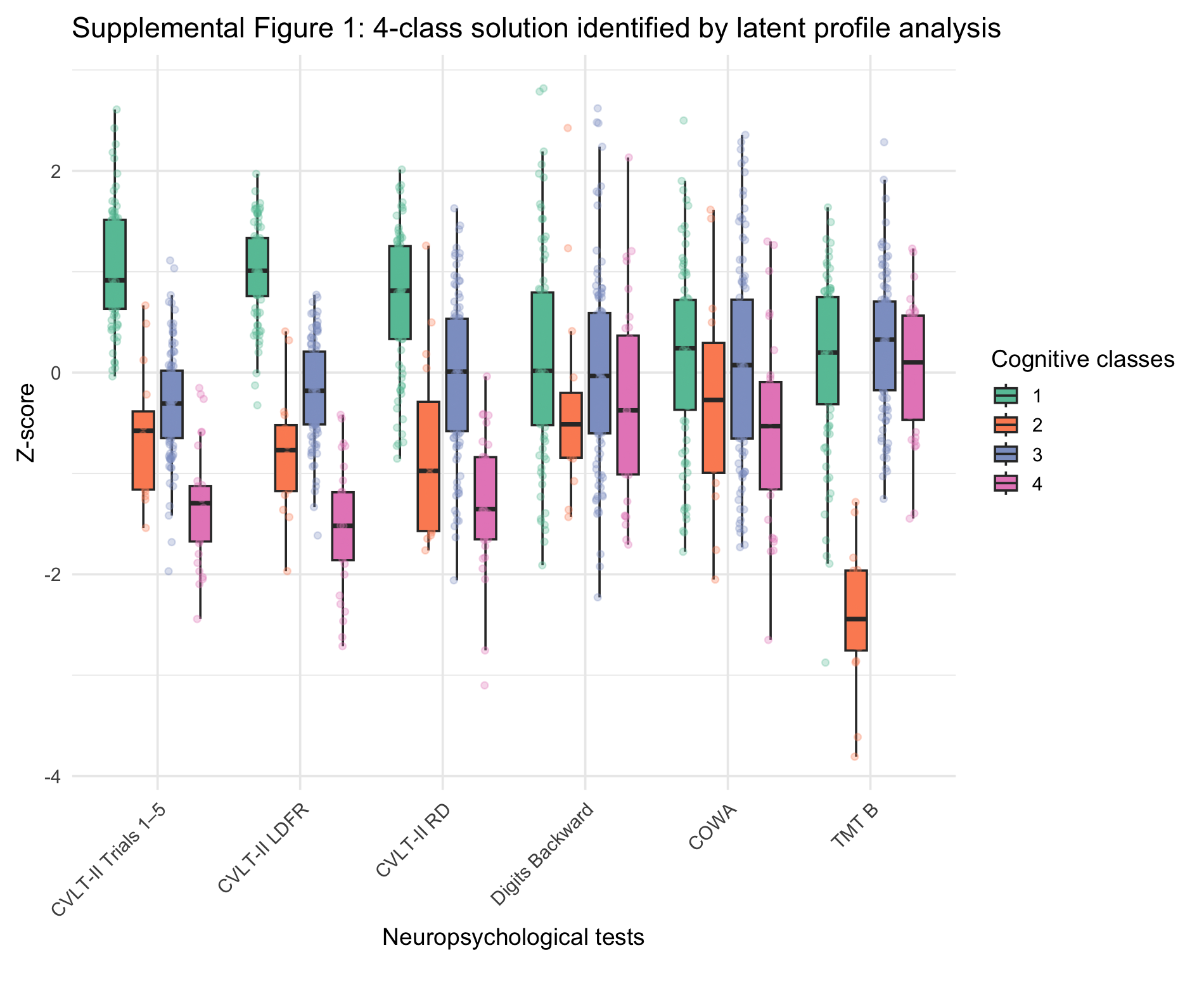


CVLT RD=California Verbal Learning Test recognition discriminability; CVLT LDFR= California Verbal Learning Test long delay free recall; CVLT 1-5= California Verbal Learning Test trials 1-5 sum; TMT B=Trail Making Test B minus A; Digits Back= Digit span backwards; COWA=Controlled Oral Word Association Total Score.

**Supplemental Figure 2: No significant differences in predicted brain age across the cognitive profile groups.**
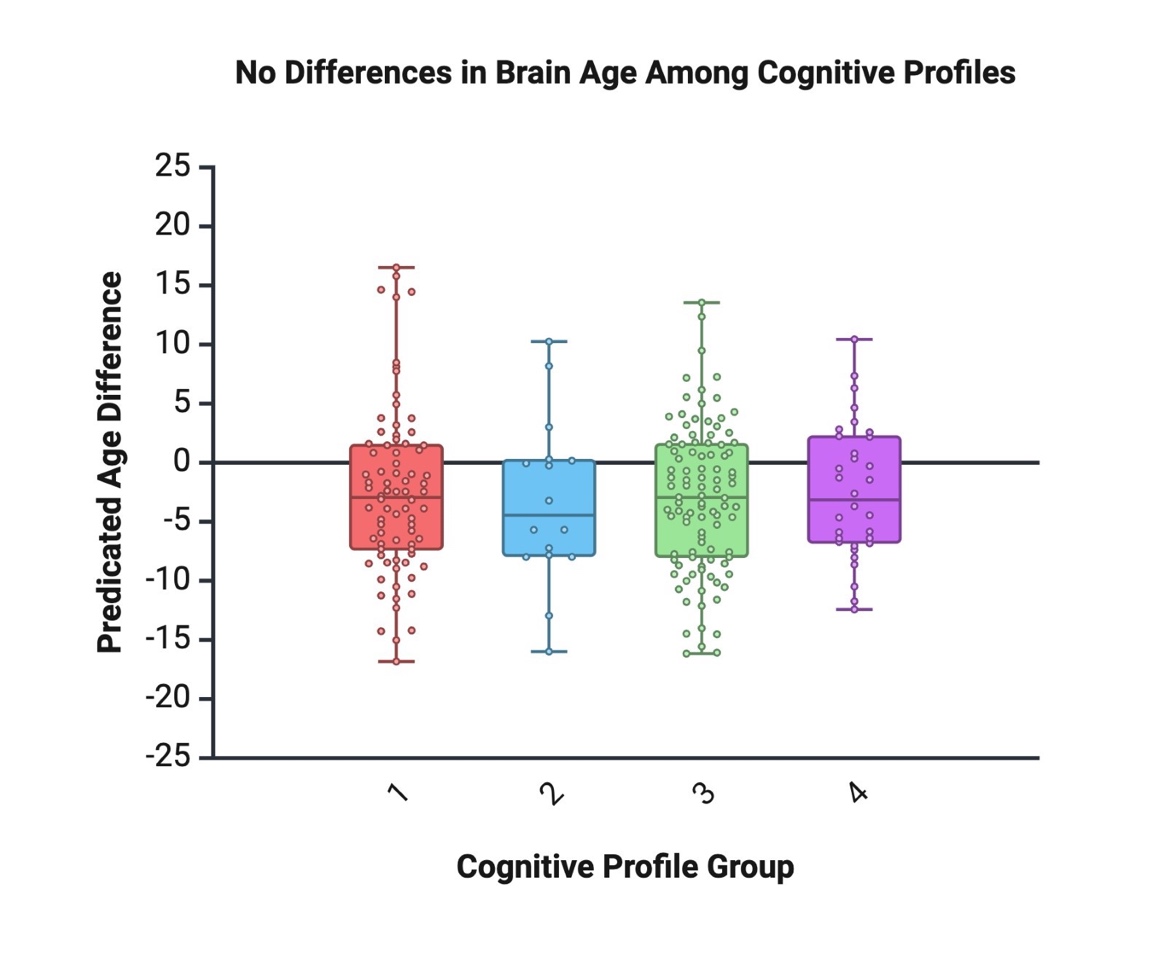


Cognitive Profile Group 1= High Memory; Cognitive Profile Group 2: Low Executive, Cognitive Profile Group 3: Global Average; Cognitive Profile Group 4: Low Memory
